# Profil des espèces fongiques isolées lors de consultations dermatologiques à Lomé (Togo)

**DOI:** 10.48327/mtsi.v4i3.2024.545

**Published:** 2024-07-15

**Authors:** Efoé SOSSOU, Ameyo DORKENOO, Akovi Kiki ADJETEY-TOGLOZOMBIO, Fiali A LACK, Atna Edi TAGBA, Azia MOUKAILA, Anoumou DAGNRA

**Affiliations:** 1 Service des laboratoires du Centre hospitalier universitaire Sylvanus Olympio, Ministère de la Santé et de l'Hygiène publique et de l'Accès universel aux soins, Quartier Tokoin hôpital, BP 57 Lomé, Togo; 2 Département de parasitologie-mycologie, Faculté des sciences de la santé, Université de Lomé, 01BP 1515 Lomé, Togo; 3 Ministère de la Santé et de l'Hygiène publique et de l'Accès universel aux soins, Avenue de la Nouvelle présidence, Centre des services administratifs BP 336 Lomé, Togo; 4 École supérieure des techniques biologiques et alimentaires (ESTBA), Université de Lomé, 01BP 1515 Lomé, Togo

**Keywords:** Mycètes, Mycoses cutanées superficielles, Contamination fongique, Consultation dermatologique, Lomé, Togo, Afrique subsaharienne, Fungi, Superficial skin mycoses, Dermatological consultation, Lomé, Togo, Sub-Saharan Africa

## Abstract

**Introduction:**

Le diagnostic de mycose cutanée superficielle (MCS) est très fréquemment évoqué lors des consultations dermatologiques en zones tropicales, mais la confirmation mycologique y est très peu réalisée en routine. L'objectif de cette étude est de décrire le profil des espèces fongiques rencontrées à l'occasion de consultations de dermatologie à Lomé (Togo), d'en établir leurs fréquences relatives et, surtout, d'en discuter leur responsabilité dans les lésions observées.

**Méthode:**

Il s'agit d'une étude descriptive réalisée de février 2020 à mars 2022. Elle a porté sur des patients présentant des lésions suspectes de MCS, reçus en consultation dermatologique dans les trois services de dermatologie de Lomé. Les prélèvements ont été réalisés sur site pour les lésions fortement suspectes de mycoses et analysés au Laboratoire de mycologie du CHU Sylvanus Olympio pour une confirmation biologique.

**Résultats:**

Au cours de la période d’étude, 565 patients ont été enrôlés pour prélèvement. Trois cent soixantequatre (64,4 %) étaient des femmes et l’âge médian était de 31 ans, avec un intervalle interquartile (IIQ) de 22 à 41 ans. L'examen direct et/ou la culture ont été positifs chez 84,7 % (479/565) d'entre eux. Les principales espèces fongiques identifiées étaient des levures du genre *Malassezia* (23,8 %), d'autres levures (39,2 %), mais également des dermatophytes (22,8 %), avec pour espèce prédominante *Trichophyton mentagrophytes* (10,8 %), et des moisissures (13,1 %) dont *Aspergillus niger* et *A. fumigatus* (3,1 % chacun). Les pseudo-dermatophytes n'ont été retrouvés que dans 1 % des cas. Des cas d'associations de mycètes ont été également notés dans 3,5 % des cas.

**Conclusion:**

Le spectre des mycètes isolés dans les suspicions de MCS à Lomé est large, mais tous ne peuvent pas être tenus pour responsables des lésions observées. Cette diversité impose donc la réalisation d'un prélèvement mycologique pour une identification précise du mycète devant toute suspicion de MCS. Selon l'espèce isolée, ceci permettra d'adapter au mieux le traitement.

## Abstract (en)

**Introduction:**

The diagnosis of superficial cutaneous mycosis (SCM) is very frequently evoked during dermatological consultations in tropical zones, but mycological confirmation is very rarely performed routinely. The aim of this study is to describe the profile of fungal species encountered during dermatology consultations in Lomé (Togo), to establish their relative frequencies and, above all, to discuss their responsibility in the lesions observed.

**Method:**

This was a descriptive study carried out from February 2020 to March 2022, involving patients presenting lesions suspected of MCS, seen in dermatological consultations. Three dermatological consultation centers, all located in the city of Lomé, served as the setting for case enrollment. Each patient consulted who met the inclusion criteria (i.e. presenting a lesion of SCM and not under antimycotic treatment or having respected an adequate therapeutic window) had a sample taken from the suspected lesion(s). Samples were taken on site and analyzed at the Mycology Laboratory of CHU Sylvanus Olympio for biological confirmation.

**Results:**

During the study period, 565 patients were enrolled and sampled, of whom 364 (64.4 %) were women. The median age was 31 years, with interquartile range (IQR) from 22 to 41 years. Direct examination and/or culture were positive in 84.7 % (479/565) of cases. The main fungal species identified were yeasts (63%), including *Malassezia sp.* (23.8%), other yeasts (39.2%), but also dermatophytes (22.8%), with *Trichophyton mentagrophytes* as the predominant species (10.8%), and molds (13.1%) including *Aspergillus niger* and *A. fumigatus* (3.1% each). Pseudo-dermatophytes were found in only 1% of cases. Fungal associations were also noted in 3.5% of cases. The most frequent associations were *C. tropicalis, A. fumigatus* and *T. mentagrophytes-C. albicans,* each found in 0.5% of cases.

**Conclusion:**

The spectrum of fungi isolated in suspected cases of SCMs in Lomé is broad, but not all can be held responsible for the lesions observed. This diversity makes it essential to take a mycological sample for accurate identification of the fungus in any suspected case of SCM. Depending on the species isolated, this will enable treatment to be tailored to the patient's needs.

## Introduction

Les mycoses sont des affections provoquées par des microorganismes appartenant au règne des *Fungi.* Elles regroupent les mycoses profondes et les mycoses superficielles. Alors que les mycoses profondes surviennent préférentiellement chez les personnes immunodéprimées, les mycoses superficielles, quant à elles, peuvent toucher aussi bien les immunocompétents que les immunodéprimés [12]. Les mycoses cutanées superficielles (MCS) font partie des affections les plus fréquentes au monde avec une prévalence variant entre 20 et 25 % [5]. Elles sont le plus souvent dues à des dermatophytes ou à des levures du genre *Malassezia* et, dans certaines circonstances, à des pseudodermatophytes, à des levures appartenant aux genres *Candida* et *Trichosporon,* ou, très rarement, à des moisissures [7, 8]. Au cours des 20 dernières années, leur incidence a augmenté de façon considérable [5, 13]. Dans les zones tropicales, ces affections sont fréquentes et souvent liées aux conditions climatiques ainsi qu’à la promiscuité [7]. Cependant, ces mêmes éléments climatiques et sociaux font que toute personne vivant en zone tropicale héberge sur la peau des espèces fongiques de l'environnement. Le lien entre « présence du champignon » et « responsabilité du champignon dans la lésion » devient ainsi difficile [1, 9].

La distribution des espèces fongiques isolées lors de suspicions de MCS est variable en fonction des pays et des populations concernées. Alors que, Ndiaye *et al.,* dans la population générale à Dakar (Sénégal), ont rapporté que les mycètes les plus identifiés étaient des levures *(Candida albicans* 26.9%) suivies des dermatophytes *(T. soudanense* 24.9% et *T. rubrum* 13,7 %) [12], Zida *et al.,* au Burkina Faso, ont trouvé une prédominance des dermatophytes (15,6 %) dominées par *T. mentagrophytes* (7 %), suivis des levures (12,2 %) majoritairement du genre *Malassezia* (11,8 %), bien que leur étude se soit déroulée en milieu carcéral [14]. Au Togo, les suspicions de MCS représentent 18,7 % des motifs de consultation des pathologies cutanées du sujet âgé [6], bien que la preuve mycologique n'ait pas toujours été apportée. Une autre étude menée par Minlekib *et al.,* 2020, [11] et portant sur une population de 135 patients, a noté une prédominance des levures (43,7 %) dominées par *M. furfur* (28,5 %), suivies des dermatophytes (20 %) avec *T. mentagrophytes* et *T. rubrum* retrouvés dans 8,7 % des cas chacun et, enfin, des moisissures (1,4 %). Mais là encore, la responsabilité exacte des mycètes de l'environnement isolés dans les lésions observées n'a pas été clairement établie. Notre présente étude, en incluant une population d’étude plus grande, se propose d'actualiser le spectre des espèces fongiques isolées dans les MCS afin d'améliorer la prise en charge thérapeutique des patients à Lomé.

## Méthods

### Type, cadre et période de l’étude

Il s'agit d'une étude descriptive menée de février 2020 à mars 2022 dans trois structures sanitaires de la ville de Lomé. Les patients reçus proviennent des trois centres qui assurent une consultation dermatologique : le Centre hospitalier universitaire Sylvanus Olympio (CHU SO) (un des trois centres de référence du niveau central de la pyramide sanitaire, son service de dermatologie réalise en moyenne 3 024 consultations par an, dont 13 % de suspicion de MCS), le centre dermatologique (CD) de Gbossimé (situé dans la même ville, il est destiné à la consultation spécialisée et reçoit en moyenne annuellement 2 722 consultations dont 19 % de suspicion de MCS) et le Centre médical des armées (CMA) de Lomé (localisé dans la partie nord de la ville et davantage orienté vers la prise en charge des cas de dermatoses chez les militaires et leurs familles). Plus d'une centaine de consultations dermatologiques y est réalisée en moyenne par an, dont 17 % de suspicion de MCS. Les prélèvements ont été faits sur site dans les centres de consultation. L'examen mycologique et l'identification des mycètes ont été faits au Laboratoire de mycologie du CHU SO, l'un des deux seuls laboratoires offrant des prestations d'analyses mycologiques à l’échelle nationale au Togo.

### Critères d'inclusion

Ont été inclus dans l’étude tous les patients présentant une lésion suspecte de MCS et n'ayant reçu aucun traitement antifongique topique dans les 15 jours ou n'ayant pas utilisé de solution filmogène sur les ongles dans les deux mois ou encore n'ayant pas été traités par un antifongique systémique dans les trois mois précédant le prélèvement et ayant donné leur consentement éclairé signé.

### Prélèvements et examens mycologiques

**Figure 1:**
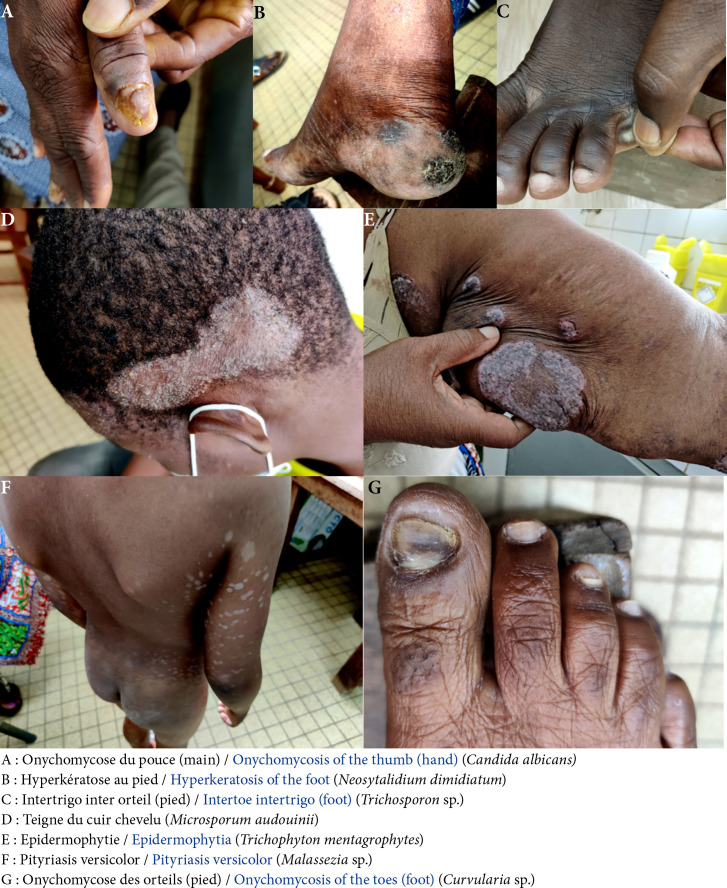
Quelques images des lésions cliniques prélevées et des champignons isolés

Les prélèvements ont été réalisés en conditions d'asepsie sur les lésions siégeant au niveau de la peau, des ongles et du cuir chevelu (Fig. 1), soit par raclage des squames sur le bord des lésions (peau), soit par coupure suivie de grattage au moyen d'un bistouri (ongle), soit par grattage des squames du cuir chevelu et arrachage à la pince des cheveux atteints. En cas de suspicion de pityriasis versicolor, le prélèvement a été fait à l'aide de cellophane adhésive, suivi d'une observation directe au microscope optique.

A : Onychomycose du pouce (main) / Onychomycosis of the thumb (hand) *(Candida albicans)*

B : Hyperkératose au pied / Hyperkeratosis of the foot *(Neosytalidium dimidiatum)*

C : Intertrigo inter orteil (pied) / Intertoe intertrigo (foot) *(Trichosporon* sp.)

D : Teigne du cuir chevelu *(Microsporum audouinii)*

E : Epidermophytie / Epidermophytia *(Trichophyton mentagrophytes)*

F : Pityriasis versicolor / Pityriasis versicolor *(Malassezia* sp.)

G : Onychomycose des orteils (pied) / Onychomycosis of the toes (foot) *(Curvularia* sp.)

Une partie de chaque échantillon prélevé a fait l'objet d'un examen direct dans une solution d'hydroxyde de potassium à 10 % ou dans une solution physiologique saline (pour les écouvillons), l'autre partie a été ensemencée sur deux milieux : Sabouraud dextrose agar (SDA) additionnée de chloramphénicol 0,5 g/l (milieu SC) et SDA additionnée de chloramphénicol 0,5 g/l et de cycloheximide 0,4 g/l (milieu SAC), les deux incubés à 25 °C [4, 7]. Les cultures ont été contrôlées visuellement tous les jours pendant 30 jours. Les espèces de mycètes ont été identifiées sur la base des résultats de l'examen direct et un ensemble de critères, notamment la vitesse de croissance, mais surtout sur les aspects macroscopique et microscopique des colonies [13]. Pour les mycètes du genre *Candida,* l'utilisation de milieux chromogéniques spécifiques (Candi sélect^®^) a permis de discriminer davantage les différentes espèces [4] et pour les mycètes du genre *Malassezia,* l'identification s'est faite à l’étape de l'examen direct.

### Recueil et analyse des données

Un formulaire spécifiquement conçu a permis de recueillir les informations sociodémographiques et cliniques des patients, de même que les résultats de l'examen mycologique. Ces données ont été saisies dans Excel 2016, puis analysées à l'aide du logiciel Epi info version 7.2.2.6. Les variables quantitatives ont été exprimées sous forme de médiane avec l'intervalle interquartile. Les tests de X^2^ et Fisher ont été utilisés pour la comparaison des données. La valeur de p < 0,05 a été considérée comme significative.

### Considérations éthiques

Cette étude a reçu l'approbation du Comité de bioéthique pour la recherche en santé du ministère de la Santé du Togo (CBRS) (N° 017/2022/ CBRS en date du 24 mai 2022) avant sa mise en œuvre. De plus, le consentement éclairé de chaque adulte inscrit et du parent ou tuteur de chaque enfant participant à l’étude a été obtenu avant leur inclusion. Les données collectées sont conservées de manière sécurisée à l'aide d'un mot de passe. Seule l’équipe d'enquête y a accès. Aucune information d'identification personnelle n'a été communiquée.

## Résultats

### Caractéristiques générales de la population d’étude

Au cours de la période d’étude, 565 patients ont été enrôlés chez qui 615 prélèvements ont été effectués. L’âge médian des patients était de 31 ans ±15,6 avec un intervalle interquartile de 22 à 41 ans, et un sex-ratio (H/F) de 0,55. La tranche d’âge [25 – 44[représentait 48,8 % (IC 95 % : 44,8 – 53). Les lésions cutanées de la peau glabre ont représenté 47,8 % (n=294) des cas, les atteintes unguéales 31,4 % (n=193) des cas, les lésions du cuir chevelu 10,2 % (n=63) des cas, l'intertrigo 8,5 % (n=52) des cas et celles des grands plis dans 2,1 % (n=13) des cas.

### Résultats des prélèvements mycologiques

Une fréquence de 84,7 % (479/565) a été notée, soit 132 (23,2 %) cas positifs uniquement à l'examen direct et 347 (61, 7 %) à l'examen direct et/ou culture; cette fréquence a varié significativement avec le centre d'enrôlement et le siège de la lésion mycosique, mais pas avec ni l’âge, ni le sexe des patients (Tableau I).

**Tableau I:** Positivité des prélèvements selon les tranches d’âges, le sexe, les centres d'enrôlement et le site des lésions

	Effectif total	Effectif positif n (%)	p
**Tranche d’âge**			
< 15	82	67 (81,7)	0,4305
[15 - 25[	87	76 (87,4)	
[25 - 45[	276	233 (84,4)	
[45 - 65[	102	89 (87,2)	
>65	18	14 (77,7)	
**Sexe**			
F	364	315 (86,5)	0,2832
M	201	159 (79,1)	
**Centres de recrutement**			
CMA Camp Gendarme-rie	54	38 (70,4)	<0,0001
CD Gbossimé	246	209 (85,1)	
Dermato CHU SO	265	232 (87,5)	
**Siège du prélèvement**			
peau glabre	294	197 (67)	0,0002
grand plis	13	12 (92,3)	
pli inter orteil	52	31 (59,6)	
ongle	193	187 (96,8)	
cheveux	63	52 (82,5)	

### Mycètes identifiés

Les mycètes les plus fréquemment isolés étaient les levures (63 % des cas), dominées par *Malassezia* sp. 23,8 % et *Candida albicans,* 14,8 %, suivis des dermatophytes dans 22,8 % des cas, avec une prédominance de *Trichophyton mentagrophytes* (11,1 %). Les pseudodermatophytes ont été isolés dans 1 % des cas, représentés par *Neoscytalidium* sp. 0,6 % et *Onychochola* sp. 0,4 % (Tableau II).

**Tableau II:** Mycètes identifiés

Mycètes identifiés	Fréquence (n)	Proportion (%)
**Levures**	**302**	**63**
*Malassezia* sp.	114	23,8
*C. albicans*	71	14,8
*C. parapsilosis*	27	5,6
*C. tropicalis*	26	5,4
*C. krusei*	21	4,3
*Candida* sp.*	17	3,5
*Trichosporon* sp.	26	5,4
**Dermatophytes**	**109**	**22,8**
*T. mentagrophytes*	52	10,8
*T. tonsurans*	18	3,7
*T. rubrum*	21	4,4
*T. violaceum*	4	0,8
*T.schoenleinii*	2	0,4
*T. soudanense*	2	0,4
*T. glabrum*	1	0,2
*Trichophyton* sp.	1	0,2
*M. audouinii*	4	0,8
*E. floccosum*	5	10
**Moisissures**	**63**	**13,1**
*A. niger*	15	3,1
*A. fumigatus*	15	3,1
*A. versicolor*	1	0,2
*A. terreus*	1	0,2
*A. nidulans*	1	0,2
*F. oxysporum*	1	0,2
*Mucor* sp.	9	1,9
*Penicilium* sp.	8	1,7
*Acremonium* sp.	4	0,8
*Fusarium* sp.	3	0,6
*Culvularia* sp.	2	0,4
*Scopuliariospis* sp.	1	0,2
*Rhizomucor* sp.	2	0,4
**Pseudo-dermatophytes**	5	1
*Neoscytalidium dimidiatum*	3	0,6
*Onychochola canadiensis*	2	0,4

C.= *Candida*; T. = *Trichophyton*; E. = *Epidermophyton*; M. = *Microsporum*; A.= *Aspergillus*; F.= *Fusarium*; *P.=Pemcillium*

* Espèce de *Candida* non identifiée par Candi sélect^®^

Des associations ont été notées dans 3,5 % des cas, les plus fréquentes étant *T. mentagrophytes C. albicans* retrouvées dans 0,5 %.

### Distribution des mycètes identifiés par tranches d’âge, par sexe et par site de prélèvement

Les dermatophytes ont été majoritairement identifiés dans les prélèvements d'ongles et du cuir chevelu avec respectivement 31,2 % et 30,3 % des cas, alors que les levures l'ont été davantage au niveau de la peau glabre (52,6 %). À part les pseudo dermatophytes, tous identifiés chez les hommes, une fréquence plus forte des autres groupes de mycètes a été notée chez les sujets de sexe féminin. De plus, la tranche d’âge [25 – 45 [a été la plus représentée pour tous les différents groupes de mycètes identifiés (Tableau III).

**Tableau III:** Distribution des mycètes identifiés par tranches d’âge, par sexe et par site de prélèvement

	Dermatophytes	Malassezia	Autres levures	Moisissures	Pseudodermatophytes
**Sexe**					
F	60 (55,1 %)	75(65,8 %)	133(70,7%)	46 (73 %)	0
M	49 (44,9 %)	39 (34,2 %)	55 (29,3%)	17 (27%)	5 (100 %)
**Tanches d’âge (ans)**					
<15	34 (31,2 %)	23 (7,6 %)	8 (7,0%)	10 (16%)	0
[15 - 25[	14 (12,8 %)	54 (17,9 %)	31 (27,2%)	8 (13 %)	0
[25 - 45[	44 (40,4 %)	161 (53,3 %)	60 (52,6%)	25 (40 %)	3 (60 %)
[45 - 65[	15 (13,8 %)	54 (17,9 %)	13 (11,4%)	19 (30 %)	1 (20 %)
>65	2 (1,8 %)	10 (3,3 %)	2 (1,8%)	1 (2 %)	1 (20 %)
**Siège de lésion**					
cuir chevelu	33 (30,3 %)	12 (4,0 %)	0	7 (11 %)	0
grands plis	3 (2,8 %)	8 (2,7 %)	0	1 (2 %)	0
ongles	34 (31,2 %)	111 (36,8 %)	0	38 (60 %)	4 (80 %)
peau glabre	22 (20,2 %)	159 (52,6 %)	114 (100,0%)	16 (25 %)	0
plis inter orteils	17 (15,6 %)	12 (4,0 %)	0	1 (2 %)	1 (20 %)

## Discussion

La présente étude actualise les données sur les mycètes isolés dans les suspicions de MCS au Togo. La forte fréquence des champignons retrouvés, soit 84,7 % des prélèvements effectués, pourrait s'expliquer par l'orientation des patients en amont de la consultation de dermatologie par les médecins privés ou publics ou les infirmiers ou d'autres professionnels de la santé ou encore par le patient lui-même. En fonction de type de mycète identifié, les levures suivies des dermatophytes puis des moisissures et des pseudodermatophytes ont été les principaux groupes fongiques retrouvés. Les types de mycètes identifiés pourraient entraîner à tort une surestimation de la prévalence des MCS retrouvée dans notre étude. En effet, dans le cas des MCS, il est essentiel de distinguer les « vraies mycoses » des « contaminations fongiques » comme l'ont démontré Chabasse *et al,* 2014 et Monod *et al,* 2013 [1, 9] puisqu'en dehors des dermatophytes et *Malassezia* sp. qui sont des mycètes absolument pathogènes, d'autres espèces peuvent parfois entraîner de réelles pathologies, mais existent plus volontiers sous la forme saprophyte ou commensale au niveau de la peau.

C'est ainsi que les *Candida* spp. et les moisissures, très présents dans l'environnement, ne peuvent être considérés responsables de mycoses que dans certaines conditions : les autres causes possibles des lésions cliniques doivent avoir été éliminées au maximum, les mycètes doivent être isolés à plusieurs reprises au niveau de la lésion, et une nette évolution clinique doit être obtenue après un traitement antifongique spécifique.

Bien que la plupart des mycètes aient été isolés à partir de lésions cutanées évocatrices, il est difficile d'affirmer avec certitude que certains d'entre eux sont des vrais pathogènes car ils n'ont été isolés qu'une seule fois à partir de lésions suspectes et que le suivi thérapeutique de ces patients n'a pas fait l'objet de notre étude. Toutefois en éliminant les mycètes identifiés comme saprophytes et environnementaux et en ne considérant que *Malassezia* sp, les dermatophytes et les pseudo dermatophytes, une fréquence révisée des MCS dans notre étude serait de 40,35 % de (228/565). Cette fréquence qui demeure toujours élevée comparativement à la moyenne mondiale comprise entre de 20 à 25% [5], pourrait être liée au climat subéquatorial qui caractérise la capitale du Togo, site de notre étude et qui favoriserait la survenue de ces MCS, puisqu'il a été démontré par d'autres études que la distribution des MCS peut être fonction du climat [2, 4, 6].

Dans les pays à faible revenu, le coût de la prise en charge des MCS est non négligeable. Cette prise en charge se fait souvent soit par une automédication, un recours à la médecine traditionnelle et dans une moindre mesure par une consultation des services de santé disponibles [8]. C'est le cas du Togo où selon la politique nationale de qualité des services de santé de 2019, environ 50 % de la population aurait recours à la médecine traditionnelle, conséquences de l'héritage culturel et de l'inaccessibilité financière et géographique aux systèmes de soins [10]. À cela, s'ajouterait, dans le cas de cette étude, la méconnaissance des mycoses par les communautés. De plus, le nombre extrêmement limité de laboratoires de mycologie dans le pays expliquerait la faible confirmation biologique des lésions suspectes de mycoses en pratique de routine. En effet, au moment de l’étude, le laboratoire de mycologie du CHU SO était le seul qui offrait des prestations de cette confirmation biologique et ce n'est qu'en juillet 2022 qu'un deuxième laboratoire a été mis en service toujours à Lomé, expliquant la forte concentration de cas au niveau de ce laboratoire. Dans le parcours des malades chez qui le diagnostic des MCS est suspecté, trois attitudes sont souvent notées, définissant ainsi trois catégories de patients. La première regroupe les patients qui, après consultation chez le spécialiste, n'ont souvent pas la possibilité de réaliser la confirmation mycologique, soit par manque de ressource financière, soit par inexistence de structure de confirmation mycologique. Dans ce cas, la prise en charge thérapeutique est probabiliste. La deuxième concerne les patients qui, après la consultation spécialisée et malgré la disponibilité des moyens financiers, ne réalisent pas la confirmation biologique, mais préfèrent un traitement par automédication ou par des médicaments traditionnels conseillés par des proches. La dernière concerne ceux qui n'ont pas confiance en la médecine occidentale et qui s'orientent directement vers les tradipraticiens pour la prise en charge de ces MCS; selon l'Organisation mondiale de la santé (OMS), cela concerne plus de 80 % des africains, particulièrement au sud du Sahara [3, 10]. Il est alors évident que, même si la fréquence de MCS retrouvée dans cette étude parait élevée, elle est sous-estimée du fait de la sous-notification des cas. De plus, comme la majorité des cas de MCS entraîne davantage de gêne esthétique et d'inconfort qu'un engagement du pronostic vital, les patients s'en accommodent et continuent à vivre avec ces lésions fongiques.

## Limites de l’étude

Le recrutement des sujets pour la présente étude étant réalisé dans les services de consultation dermatologiques tous situés à Lomé, nos résultats ne peuvent donc pas être extrapolés à tout le pays. Par ailleurs, certains participants pourraient avoir été inclus à tort, le critère d'inclusion concernant l'absence de médication antifongique récente n'ayant été vérifié que de manière déclarative. De plus, il a été difficile de séparer les vraies MCS des cas de mycètes saprophytes ou commensaux isolés. Enfin, notre travail a été réalisé dans un contexte de plateau technique de routine qui ne garantit pas l'identification des espèces rares ou émergentes. Ces éléments pourraient influencer nos résultats.

## Conclusion

La présente étude a permis d'avoir une idée plus large de l'ensemble des mycètes isolés à partir des lésions présentées par les patients et de s'interroger sur la proportion des vrais mycètes pathogènes recueillis comparativement aux simples champignons saprophytes de la peau.

Une forte fréquence des MCS a été cependant observée dans la population d’étude avec un spectre dominé par des levures du genre Malassezia suivies des dermatophytes, *T. mentagrophytes* étant l'espèce de dermatophytes prépondérante. Une sensibilisation de la population serait très utile à travers des émissions radiodiffusées ou télévisées sur la nécessité de confirmer les lésions dermatologiques suspectes par des examens mycologiques de laboratoire. Ceci permettrait en particulier de réserver les traitements antifongiques, souvent onéreux, aux « vraies mycoses ». Le problème d'accessibilité financière limitée des populations constituant le frein à la réalisation de ces examens de laboratoires, serait levé grâce à l'assurance maladie universelle mise en œuvre tout récemment au Togo. Il est également important de mettre aux normes le plateau technique et d’étendre ces laboratoires de mycologie à l’échelle nationale ce qui aiderait à statuer davantage sur la pathogénicité de mycètes commensaux identifiés dans notre série. Il est donc urgent studies to refine our frequencies and the spectrum of commensal mycetes implicated in SCM in the country. This will ensure the acquisition of upto-date national data on the prevalence of SCM.

## Financement

Cette étude a été réalisée sur financement personnel.

## Remerciements

Nos remerciements à tous les étudiants qui ont aidé à la réalisation des différents prélèvements dans les centres d'enrôlement des patients, sans oublier les anonymes qui ont contribué à l'amélioration du manuscrit par leur lecture critique.

## Contribution des auteurs

Efoé SOSSOU : rédaction du protocole, réalisation des prélèvements et des analyses de laboratoire, rédacteur principal du manuscrit, correspondant de l’étude. Ameyo DORKENOO : conception de l’étude, validation du protocole, lecture et correction du manuscrit; Fiali A. LACK, Atna Edi TAGBA, Azia MOUKAILA : relecture et correction du manuscrit; Akovi Kiki ADJETEYTOGLOZOMBIO : contribution à l'amélioration du manuscrit. Anoumou DAGNRA : coordination et validation de la version finale du manuscrit.

## Déclaration d'intérêt

Les auteurs ne rapportent aucun conflit d'intérêt. Les auteurs sont seuls responsables du contenu et de la rédaction de l'article.

## References

[1] Chabasse D, Pihet M (2014). Méthodes de diagnostic d'une onychomycose. J Mycol Med.

[2] Chandenier J, Desoubeaux G (2015). La transition épidémiologique des mycoses en Afrique subsaharienne : de la surface vers la profondeur. Bull Soc Pathol Exot.

[3] Doumbia H (2015). Place de la médecine traditionnelle dans la prise en charge thérapeutique des enfants de moins de 5 ans avant leur hospitalisation au CS Réf de Koutiala. Thèse Doc Médecine, Univ. Bamako.

[4] Eloy O, Blanc V, Mallié M, Decousser JW, Pina P, Allouch PY (2005). Identification et sensibilité aux antifongiques de deux souches de *Candida* dans 95 hôpitaux français. J Mycol Med.

[5] Ezomike NE, Ikefuna AN, Onyekonwu CL, Ubesie AC, Ojinmah UR, Ibe BC (2021). Epidemiology and pattern of superficial fungal infections among primary school children in Enugu, south-east Nigeria. Malawi Med J.

[6] Kombaté K, Saka B, Mouhari-Toure A, Barruet RK, Gnassingbé W, Akakpo S, Mabou-dou A, Landoh DE, Tchangaï-Walla K, Pitché P (2014). Pathologie cutanée du sujet âgé en dermatologie à Lomé, Togo: étude de 325 cas. Pan Afr Med J.

[7] Lipner SR, Scher RK (2019). Onychomycosis: Clinical overview and diagnosis. J Am Acad Dermatol.

[8] Mahé A, Faye O, Fanello S (2003). Dermatologie et santé publique dans les pays en voie de développement. Bull Soc Pathol Exot.

[9] Monod M, Lurati M, Baudraz-Rosselet F (2013). Diagnostic des onychomycoses à moisissures et importance pour le traitement. Rev Med Suisse.

[10] Ministère de la santé et de l'hygiène publique (2019). Politique nationale de qualité des services de santé au Togo.

[11] Minlekib PC, Dorkenoo AM, Sow D, Dia M, Manga IA, Fall CB, Lelo S, Sossou E, Sylla K, Ndiaye M, Ndiaye JL, Tine RC, Dieng T, Faye B (2021). Epidemiology of Superficial Fungal Infections in Hospital Settings in Togo and Senegal from 2019 to 2020. Open Access J Mycol Mycol Sci.

[12] Ndiaye M, Diongue K, Sadikh Badiane A, Cheikh Seck M, Ndiaye D (2017). Profil épidémiolo-gique des mycoses superficielles isoles à Dakar. Étude rétrospective de 2011 à 2015. J Med Mycol.

[13] Oussou MA, Gue I, Zika KD, Kouassi KA, Kassi K (2020). Teignes en milieu scolaire primaire à Bouaké - Côte d'Ivoire : Aspects épidémiologiques, cliniques et microbiologiques. Rev Int Sc Méd ABJ.

[14] Zida A, Barro-Traoré F, Dera M, Bazié Z, Niamba P, Guiguemdé TR (2015). Aspects épidémiologiques et étiologiques des mycoses cutanéophanériennes chez les détenus de la maison d'arrêt et de correction d'Ouagadougou (Burkina Faso). J Mycol Med.

