# Supplementary material for: Profile of fungal species involved in superficial cutaneous mycoses in Lomé (Togo)
Source: Med Trop Sante Int. 2024 Jul 15;4(3):mtsi.v4i3.2024.545. [Article in French] doi: 10.48327/mtsi.v4i3.2024.545 (PMC11809061; doi:10.48327/mtsi.v4i3.2024.545)
Supplement: Related texts

# Research article: Profile of fungal species involved in superficial cutaneous mycoses in Lomé (Togo)

## Introduction

Mycoses are diseases caused by microorganisms belonging to the kingdom of fungi. There are deep and superficial mycoses. While deep mycoses occur preferentially in immunocompromised individuals, superficial mycoses can affect both immunocompetent and immunocompromised individuals [12]. Superficial cutaneous mycoses (SCM) are among the most common diseases worldwide, with a prevalence of 20% to 25% [5]. They are most commonly caused by dermatophytes or yeasts of the genus *Malassezia* and, in certain circumstances, by pseudodermatophytes, yeasts of the genera *Candida* and *Trichosporon* or, very rarely, molds [7, 8]. The incidence of these diseases has increased significantly in the last 20 years [5, 13]. In tropical areas, these diseases are common and often associated with climatic conditions and promiscuity [7]. However, these same climatic and social factors mean that anyone living in a tropical zone will harbor environmental fungal species on their skin. The relationship between “presence of fungus” and “responsibility of the fungus for the lesion” is therefore difficult to establish [1, 9].

The distribution of fungal species isolated in cases of suspected SCM varies significantly between countries and populations. In the general population of Dakar (Senegal), Ndiaye *et al.* reported that the most commonly identified fungi were yeasts *(Candida albicans* 26.9%), followed by dermatophytes *(T. soudanense* 24.9% and *T. rubrum* 13.7%) [12]. In a study conducted in Burkina Faso, Ndiaye *et al.* observed a prevalence of dermatophytes (15.6%) predominantly represented by *T. mentagrophytes* (7%), followed by yeasts (12.2%) predominantly belonging to the *Malassezia* genus (11.8%). However, it is noteworthy that their study was conducted in a prison setting [14].

In Togo, suspicion of SCM accounts for 18.7% of consultations for skin pathologies in the elderly [6], although mycological evidence has not always been provided. Another study by Minlekib *et al,* 2020, [11] involving a population of 135 patients, noted a predominance of yeasts (43.7%), dominated by *M. furfur* (28.5%), followed by dermatophytes (20%), with *T. mentagrophytes* and *T. rubrum* found in 8.7% of cases each, and finally molds (1.4%). However, the precise role of environmental fungi isolated from the lesions remains unclear. Our present study, which includes a larger study population, aims to update the spectrum of fungal species isolated in SCM in order to improve the therapeutic management of patients in Lomé.

## Methods

### Type, scope and period of study

This descriptive study was conducted from February 2020 to March 2022 in three health facilities in the city of Lomé. Patients were recruited from three dermatological consultation centers. The Center hospitalier universitaire Sylvanus Olympio (CHU SO) (one of three reference centers at the central level of the health pyramid. Its dermatology department carries out an average of 3,024 consultations per year, 13% of which are for suspected SCM), the Centre dermatologique (CD) de Gbossimé (located in the same city, it is used for specialized consultations and receives an average of 2,722 consultations a year, 19% of which are for suspected SCM) and the Centre Médical des Armées (CMA) in Lomé (located in the northern part of the city, and geared more towards treating dermatitis in military personnel and their families). On average, more than a hundred dermatological consultations are conducted annually at this facility, with 17% of these cases involving suspected SCM. Samples were taken on site at the consultation centers. Mycological examination and identification were carried out at the Mycology Laboratory of the CHU SO, one of only two laboratories offering mycological analysis services nationwide in Togo.

### Inclusion criteria

All patients with a suspected SCM lesion who had not received topical antifungal treatment within 15 days, or who had not used a film-forming nail solution within two months, or who had not been treated with a systemic antifungal agent within three months prior to sampling, and who had provided signed informed consent, were included in the study.

### Mycological sampling and examination

Samples were obtained from lesions on the skin, nails, and scalp (Fig. 1) under aseptic conditions. The samples were taken either by scraping the scales from the edge of the lesion (skin), by cutting and scraping with a scalpel (nails), or by scraping the scales from the scalp and plucking the affected hair with forceps. In cases of suspected pityriasis versicolor, samples were taken using adhesive cellophane, followed by direct observation under a light microscope.

**Figure 1:** Some images of the clinical lesions sampled and fungi isolated

A : Onychomycose du pouce (main) / Onychomycosis of the thumb (hand) *(Candida albicans)*

B : Hyperkératose au pied / Hyperkeratosis of the foot *(Neosytalidium dimidiatum)*

C : Intertrigo inter orteil (pied) / Intertoe intertrigo (foot) *(Trichosporon* sp.)

D : Teigne du cuir chevelu *(Microsporum audouinii)*

E : Epidermophytie / Epidermophytia *(Trichophyton mentagrophytes)*

F : Pityriasis versicolor / Pityriasis versicolor *(Malassezia* sp.)

G : Onychomycose des orteils (pied) / Onychomycosis of the toes (foot) *(Curvularia* sp.)

Part of each sample taken was examined directly in 10% potassium hydroxide solution or in physiological saline solution (for swabs), while the other part was inoculated on two media: Sabouraud dextrose agar (SDA) supplemented with chloramphenicol 0.5 g/l (SC medium) and SDA supplemented with chloramphenicol 0.5 g/l and cycloheximide 0.4 g/l (SAC medium), both incubated at 25°C [4, 7]. Cultures were checked visually every day for 30 days. Fungal species were identified on the basis of the results of direct examination and a set of criteria, notably growth rate, but above all on the macroscopic and microscopic aspects of the colonies [13]. For fungi of the *Candida* genus, the use of specific chromogenic media (Candi sélect^®^) has enabled further discrimination between the different species [4], while for fungi of the *Malassezia* genus, identification was made at the direct examination stage.

### Data collection and analysis

A specially designed form was used to collect patients’ sociodemographic and clinical information, as well as the results of the mycological examination. These data were entered into Excel 2016, then analyzed using Epi info software version 7.2.2.6. Quantitative variables were expressed as median with interquartile range. The X^2^ and Fisher tests were used to compare data. A value of p < 0.05 was considered significant.

### Ethical considerations

Prior to implementation, the study received approval from the Togo Ministry of Health's Bioethics Committee for Health Research (CBRS) (No. 017/2022/CBRS, May 24, 2022). Furthermore, informed consent was obtained from each enrolled adult and from the parent or guardian of each child participating in the study prior to their inclusion. The data collected is stored in a secure, password-protected environment, accessible only to members of the survey team. No personally identifying information has been disclosed.

## Results

### General characteristics of the study population

A total of 565 patients were enrolled in the study, and 615 samples were collected from them. The median age of patients was 31 ± 15.6 years, with an interquartile range of 22 to 41 years, and a sex ratio (M/F) of 0.55. The age range [25 - 44[accounted for 48.8% (95% CI: 44.8 - 53). The most prevalent lesions were those affecting the hairless skin (47.8%, n=294), followed by nail lesions (31.4%, n=193), scalp lesions (10.2%, n=63), intertrigo (8.5%, n=52), and fold lesions (2.1%, n=13).

### Mycological sampling results

A frequency of 84.7% (479/565) was observed, indicating that 132 cases (23.2%) were positive only upon direct examination and 347 cases (61.7%) were positive upon direct examination and/or culture. This frequency exhibited notable variation across different enrollment centers and sites of mycotic lesions. However, it remained consistent with regard to the age and sex of the patients (Table I).

**Table I:** Samples positivity by age group, sex, enrollment centers and site of lesions

	Total population	Number of positive n	p
**Age range**			
< 15	82	67 (81,7)	0,4305
[15 - 25[	87	76 (87,4)	
[25 - 45[	276	233 (84,4)	
[45 - 65[	102	89 (87,2)	
>65	18	14 (77,7)	
**Sex**			
F	364	315 (86,5)	0,2832
M	201	159 (79,1)	
**Recruitment centers**			
CMA Camp Gendarme-rie	54	38 (70,4)	<0,0001
CD Gbossimé	246	209 (85,1)	
Dermato CHU SO	265	232 (87,5)	
**Sampling site**			
hairless skin	294	197 (67)	0,0002
large skin folds	13	12 (92,3)	
inter-toe crease	52	31 (59,6)	
nail	193	187 (96,8)	
hair	63	52 (82,5)	

### Identified mycetes

The most frequently isolated fungi were yeasts (63% of cases), with *Malassezia* sp. and *Candida albicans* representing 23.8% and 14.8% of cases, respectively. Dermatophytes were the second most common fungi, accounting for 22.8% of cases, with *Trichophyton mentagrophytes* being the predominant species (11.1%). Pseudodermatophytes were isolated in 1% of cases, with 0.6% representing *Neoscytalidium* sp. and 0.4% representing *Onychocola* sp. (Table II).

**Table II:** Identified fungi

Mycètes identifiés	Frequency (n)	Proportion (%)
**Yeast**	**302**	**63**
*Malassezia* sp.	114	23,8
*C. albicans*	71	14,8
*C. parapsilosis*	27	5,6
*C. tropicalis*	26	5,4
*C. krusei*	21	4,3
*Candida* sp.*	17	3,5
*Trichosporon* sp.	26	5,4
**Dermatophytes**	**109**	**22,8**
*T. mentagrophytes*	52	10,8
*T. tonsurans*	18	3,7
*T. rubrum*	21	4,4
*T. violaceum*	4	0,8
*T.schoenleinii*	2	0,4
*T. soudanense*	2	0,4
*T. glabrum*	1	0,2
*Trichophyton* sp.	1	0,2
*M. audouinii*	4	0,8
*E. floccosum*	5	10
**Molds**	**63**	**13,1**
*A. niger*	15	3,1
*A. fumigatus*	15	3,1
*A. versicolor*	1	0,2
*A. terreus*	1	0,2
*A. nidulans*	1	0,2
*F. oxysporum*	1	0,2
*Mucor* sp.	9	1,9
*Penicilium* sp.	8	1,7
*Acremonium* sp.	4	0,8
*Fusarium* sp.	3	0,6
*Culvularia* sp.	2	0,4
*Scopuliariospis* sp.	1	0,2
*Rhizomucor* sp.	2	0,4
**Pseudo-dermatophytes**	5	1
*Neoscytalidium dimidiatum*	3	0,6
*Onychochola canadiensis*	2	0,4

C.= *Candida*; T. = *Trichophyton*; E. = *Epidermophyton*; M. = *Microsporum*; A.= *Aspergillus*; F.= *Fusarium*; *P.=Pemcillium*

* *Candida* species not identified by Candi sélect^®^

Associations were noted in 3.5% of cases, the most frequent being *T. mentagrophytes-C. albicans* found in 0.5%.

### Distribution of identified mycetes by age group, sex and sampling site

Dermatophytes were predominantly identified in nail and scalp samples (31.2% and 30.3% respectively), while yeasts were identified more frequently in hairless skin (52.6%). With the exception of pseudodermatophytes, all of which were identified in men, a higher frequency of other fungal groups was noted in female subjects. In addition, the age range [25 - 45[was the most prevalent among all identified fungal groups (Table III).

**Table III:** Distribution of fungi identified by age groups, by sex and by sampling site

	Dermatophytes (n=109)	Malassezia (n=114)	Other yeast species (n=188)	Molds (n=63)	Pseudodermatophytes (n=5)
**Sex**					
F	60 (55,1 %)	75(65,8 %)	133(70,7%)	46 (73 %)	0
M	49 (44,9 %)	39 (34,2 %)	55 (29,3%)	17 (27%)	5 (100 %)
**Age range (years)**					
<15	34 (31,2 %)	23 (7,6 %)	8 (7,0%)	10 (16%)	0
[15 - 25[	14 (12,8 %)	54 (17,9 %)	31 (27,2%)	8 (13 %)	0
[25 - 45[	44 (40,4 %)	161 (53,3 %)	60 (52,6%)	25 (40 %)	3 (60 %)
[45 - 65[	15 (13,8 %)	54 (17,9 %)	13 (11,4%)	19 (30 %)	1 (20 %)
>65	2 (1,8 %)	10 (3,3 %)	2 (1,8%)	1 (2 %)	1 (20 %)
**Site of lesion/sampling**					
scalp	33 (30,3 %)	12 (4,0 %)	0	7 (11 %)	0
large skin folds	3 (2,8 %)	8 (2,7 %)	0	1 (2 %)	0
nails	34 (31,2 %)	111 (36,8 %)	0	38 (60 %)	4 (80 %)
hairless skin	22 (20,2 %)	159 (52,6 %)	114 (100,0%)	16 (25 %)	0
inter-toe crease	17 (15,6 %)	12 (4,0 %)	0	1 (2 %)	1 (20 %)

## Discussion

The present study updates data on fungi isolated from suspected cases of SCM in Togo. The high frequency of fungi found, i.e. in 84.7% of the samples taken, could be explained by the referral of patients prior to dermatological consultation by private or public doctors, nurses or other health professionals, or by the patient himself. According to the type of fungus identified, yeasts, followed by dermatophytes, then molds and pseudodermatophytes were the main fungal groups found. The types of fungi identified could erroneously lead to an overestimation of the prevalence of SCM found in our study. In fact, in the case of SCM, it is essential to distinguish between “true mycoses” and “fungal contaminations”, as shown by Chabasse *et al*, 2014 and Monod *et al*, 2013 [1, 9], because apart from dermatophytes and *Malassezia* sp., which are absolutely pathogenic fungi, other species can sometimes cause real pathologies, but are more likely to exist in saprophytic or commensal form on the skin. Thus, *Candida* spp. and molds, which are very present in the environment, can only be considered responsible for mycoses under certain conditions: other possible causes of clinical lesions must have been eliminated as far as possible, the fungi must be isolated repeatedly from the lesion, and a clear clinical evolution must be obtained after specific antifungal treatment.

Although the majority of the fungi were isolated from suggestive skin lesions, it is challenging to definitively ascertain that some of them are true pathogens, as they were only isolated once from suspicious lesions and the therapeutic follow-up of these patients was not the subject of our study. However, by eliminating mycoses identified as saprophytic and environmental, by considering only *Malassezia* sp., dermatophytes, and pseudodermatophytes, a revised frequency of SCM in our study would be 40.35% (228/565) of the total number of cases. This frequency remains elevated in comparison to the global average of 20 to 25% [5], and may be attributed to the sub-equatorial climate of the capital of Togo, the site of our study, which is conducive to the proliferation of SCM. This assertion is supported by the findings of other studies which have demonstrated that the distribution of SCM can be influenced by climate [2, 4, 6].

In low-income countries, the financial burden associated with the management of SCM is a significant concern. This is frequently accomplished through self-medication, the utilization of traditional medicine, and, to a lesser extent, the consultation of available health services [8]. This is exemplified in Togo, where, as per the 2019 national health service quality policy, approximately 50% of the population relies on traditional medicine, due to cultural heritage and financial and geographical inaccessibility to healthcare systems [10]. Furthermore, this study is limited by the lack of knowledge about mycoses among the communities under investigation. Furthermore, the dearth of mycology laboratories in the country contributes to the low prevalence of biological confirmation of suspected mycosis lesions in routine practice. At the time of the study, the mycology laboratory of the CHU SO was the sole facility offering such services, and a second laboratory was only commissioned in Lomé in July 2022, which explains the high concentration of cases at this laboratory.

In the case of patients with suspected SCM, three attitudes are often observed, which define three categories of patient. The initial category encompasses patients who, subsequent to a consultation with a specialist, frequently lack the financial resources or the availability of mycological confirmation facilities, thereby precluding the possibility of mycological confirmation. In this case, therapeutic management is probabilistic. The second category of patients is comprised of those who, despite the availability of financial resources and the recommendation of a specialist, choose not to undergo biological confirmation and instead opt for self-medication or traditional medicines recommended by relatives. The last concerns those who have no confidence in Western medicine and who turn directly to traditional practitioners for the treatment of SCDs; according to the World Health Organization (WHO), this concerns over 80% of Africans, particularly those living south of the Sahara [3, 10]. Therefore, although the frequency of SCM found in this study appears high, it is likely to be an underestimate due to under-reporting of cases. Furthermore, as the majority of cases of SCM involve more aesthetic discomfort than life-threatening disease, patients are able to live with the fungal lesions.

## Study limits

As the subjects for the present study were recruited from dermatological consultation departments, all of which were located in Lomé, it is not possible to extrapolate the results to the whole country. In addition, it is possible that some participants may have been included incorrectly, as the inclusion criterion concerning the absence of recent antifungal medication was only verified declaratively. Furthermore, it was challenging to differentiate between true SCM and cases of isolated saprophytic or commensal fungi. Finally, it should be noted that our work was carried out in the context of a routine technical platform, which does not guarantee the identification of rare or emerging species. These factors could potentially influence our results.

## Conclusion

The present study provided a more comprehensive understanding of the mycetes isolated from the lesions presented by patients, prompting inquiries regarding the proportion of true pathogenic mycetes collected compared to simple saprophytic fungi of the skin.

However, a high frequency of SCM was observed in the study population, with a spectrum dominated by yeasts of the genus *Malassezia,* followed by dermatophytes. The predominant dermatophyte species was *T. mentagrophytes.*

It would be beneficial to raise public awareness of the necessity to confirm suspicious dermatological lesions with mycological laboratory tests through radio or television broadcasts. In particular, this would facilitate the reservation of costly antifungal treatments for “real mycoses.” The issue of limited financial accessibility for the population, which presents a barrier to the performance of these laboratory tests, could be addressed through the implementation of a universal health insurance scheme, as recently introduced in Togo. It is also crucial to enhance the technical capabilities of these mycology laboratories and to expand their reach nationwide. This would facilitate the clarification of the pathogenicity of the commensal fungi identified in our series. Consequently, it is imperative to promptly commence further d'initier d'autres études permettant d'affiner nos fréquences obtenues de même que le spectre des mycètes impliqués dans les MCS dans le pays en vue de disposer de données actualisées à l’échelle nationale sur la prévalence des MCS.

## Financing

This study was carried out with personal financing.

## Acknowledgements

We would like to express our gratitude to all the students who assisted with the various sampling operations at the patient enrollment centers. We would also like to acknowledge the anonymous contributors who provided valuable feedback on the manuscript through their critical reading.

## Authors’ contributions

Efoé SOSSOU: drafting of the protocol, sampling and laboratory analysis, main editor of the manuscript, study correspondent. Ameyo DORKENOO: conception of the study, validation of the protocol, reading and correction of the manuscript; Fiali A. LACK, Atna Edi TAGBA, Azia MOUKAILA: proofreading and correction of the manuscript; Akovi Kiki ADJETEYTOGLOZOMBIO: contribution to the improvement of the manuscript. Anoumou DAGNRA: coordination and validation of the final version of the manuscript.

## Declaration of interest

The authors declare no conflict of interest. The authors are solely responsible for the content and writing of the article.
